# Prevalence and genotyping distribution of *Enterocytozoon bieneusi* in diarrheic pigs in Chongqing and Sichuan provinces, China

**DOI:** 10.3389/fmicb.2022.1025613

**Published:** 2022-10-13

**Authors:** Samson Teweldeberhan Ghebremichael, Xianzhi Meng, Junhong Wei, Yujiao Yang, Qingyuan Huang, Lie Luo, Heng Xiang, Jie Chen, M. A. Abo-Kadoum, Tian Li, Xiao Liu, Jialing Bao, Zeyang Zhou, Guoqing Pan

**Affiliations:** ^1^State Key Laboratory of Silkworm Genome Biology, Southwest University, Chongqing, China; ^2^Chongqing Key Laboratory of Microsporidia Infection and Control, Southwest University, Chongqing, China; ^3^Department of Biology, Mai Nefhi College of Science, Mai-Nefhi, Eritrea; ^4^College of Veterinary Medicine, Southwest University, Chongqing, China; ^5^College of Animal Science and Technology, Southwest University, Chongqing, China; ^6^Botany and Microbiology Department, Faculty of Science, Al-Azhar University, Assuit Branch, Cairo, Egypt; ^7^College of Life Sciences, Chongqing Normal University, Chongqing, China

**Keywords:** prevalence, *Enterocytozoon bieneusi*, genotyping, diarrheic pigs, internal transcribed spacer, novel

## Abstract

The microsporidian fungal pathogen *Enterocytozoon bieneusi* is a unicellular parasite that infects humans and various animals, including pigs. Currently, there are few data on *E. bieneusi* infection a in diarrheic pigs in Chongqing and Sichuan Provinces, China. This study aims to determine the prevalence and genotype distribution of *E. bieneusi* in diarrheic pigs. In total, 514 fecal samples from diarrheic pigs were obtained from 14 large-scale farms in Chongqing and Sichuan Provinces (326 suckling pigs, 17 weaned pigs, 65 fattening pigs, and 106 sows). To identify the *E. bieneusi* genotypes, genomic DNA was isolated from the samples and tested by nested PCR, targeting the internal transcribed spacer region of the rRNA followed by DNA sequence analysis. The overall prevalence of *E. bieneusi* was 79.8% (410/514), with rates of 84.9% (90/106) in sows and 64.7% (11/17) in weaned pigs. We found 61 different genotypes, including seven known genotypes (E, F, CHG1, Peru8, CAF1, B, and BEB17) and 54 novel genotypes. These 54 new genotypes are variants of eight known genotypes (SDD2, A, B, HLJD-IV, PigSpEb1, O, JLD-I, and BEB17) based on their sequence similarities. Phylogenetically, all of the identified genotypes clustered with counterparts belonging to Group 1 and Group 2 of *E. bieneusi*. Therefore, we found a higher prevalence of *E. bieneusi* in sows than in preweaned and weaned pigs. These findings indicate that diarrheic pigs could be a potential reservoir host, which can contaminate the environment and be a source of microsporidia in humans and other animals.

## Introduction

Diarrhea is a common disease in pigs, especially in piglets worldwide (Stentiford et al., 2016). Diarrhea can be caused by different enteropathogens (bacteria, viruses, parasites, and microsporidia). It is naturally transmitted from the asymptomatic carrier and symptomatic pigs to susceptible animals (Gyles and Fairbrother, 2010; Dubreuil et al., 2016). It is a leading cause of death in pigs as a global problem (Dubreuil et al., 2016). Currently, 17 known species of microsporidia can infect humans, the most predominant of which is *Enterocytozoon bieneusi* (Li et al., 2020; Liu et al., 2021). *E. bieneusi* is an obligate intracellular zoonotic fungus that infects various invertebrate and vertebrate hosts (Li et al., 2019b) and lives in the digestive tract. It can parasitize the host’s enterocytes, resulting in self-limiting diarrhea in humans and animals. Moreover, *E. bieneusi* can cause life-threatening chronic diarrhea in immunodeficient patients, such as AIDS patients, organ transplant recipients, and diabetes patients (Didier and Weiss, 2011). It can also promote abnormal development and growth in young animals (Zou et al., 2019). Pigs are one of the primary reservoir hosts for *E. bieneusi*. The prevalence of *E. bieneusi* infection in China was higher in pigs aged <1 month (63.6%) than in pigs aged 1–2 months (41.0%) and > 2 months (26.3%; Li et al., 2014a). *E. bieneusi* is mainly transmitted by the fecal-oral route through ingestion of contaminated water or food or accidental ingestion of spores excreted in the feces of infected animals or humans (Stentiford et al., 2016). The spores of this pathogen released into the environment by both symptomatic and asymptomatic hosts can also be a significant source of watery microsporidiosis outbreaks (Wang et al., 2018c).

Because the spores of *E. bieneusi* are very small (1 μm), it is difficult to differentiate them from the spores of other species of microsporidia using conventional staining techniques (Santín and Fayer, 2011).Therefore, PCR and ribosomal internal transcribed spacer (ITS) nucleotide sequence polymorphisms are widely used for the molecular typing of *E. bieneusi* (Karim et al., 2014; Li et al., 2019b). The ITS of rRNA exhibits substantial genetic diversity among isolates, allowing for the differentiation of host-specific and zoonotic genotypes (Amer et al., 2019). ITS genotyping has been responsible for detecting more than 500 different genotypes of *E. bieneusi*. Among these, 142 genotypes have been discovered in humans, more than 139 genotypes in pigs or wild boars worldwide, and 49 genotypes have been detected in humans and animals (Zhou et al., 2020; Dashti et al., 2022). Out of the 139 ITS genotypes identified in pigs or wild boars, 19 genotypes (CHN1, Bfrmr2, CAF1, CS-1, CS-4, D, EbpA, EbpC, EbpD, H, Henan-III Henan-IV, I, LW1, O, PigEBITS5, PigEBITS7, PigEB10, SH8) have been identified in humans (Zhou et al., 2020). Based on the phylogenetic analyses of ITS sequences, these identified *E. bieneusi* genotypes can be classified into at least 11 phylogenetic groups (Groups 1 to 11). Group 1 is a broad group intimately associated with the zoonotic potential for microsporidiosis and is primarily found in humans and animals (Liu et al., 2021). Group 2 has also recently shown increased zoonotic transmission potential recently, with the genotypes BEB4, BEB6, I, and J identified in cattle, deer, sheep, and goats. However, genotypes in Groups 3–11 are more host-specific (Li et al., 2020; Liu et al., 2021). In addition, all pig genotypes fall into one of two groups: Group 1 (94. 9%,132/139) or Group 2 (5. 0%, 7/139), meaning that pigs play a crucial role in *E. bieneusi* epidemiology as a reservoir host (Li et al., 2019c; Zhou et al., 2020; Dashti et al., 2022).

The pig industry is a significant economic contributor in China, where humans and pigs live in close areas; thus, disease can spread quickly, potentially devastatingly impacting the economy (Zhou et al., 2020). Chongqing and Sichuan are large provinces in southwest China located near the upper Yangtze River. They have a sizeable pig production industry (1,121,000 tons in 2019) combined with the highest *per capita* consumption of pork in China (33.7 kg in Chongqing versus 20.3 kg in the whole country in 2019; 2020 China Statistical Yearbook; Zhang et al., 2021). In southwest China (Chongqing and Sichuan), there is a lack of data on the prevalence and genetic diversity of *E. bieneusi* infection in diarrheic pigs, except for a few studies in asymptomatic Tibetan pigs (Luo et al., 2019), wild boar (Li et al., 2017), masked palm civets (Yu et al., 2020), pet birds (Deng et al., 2019), pet rabbits (Deng et al., 2020b), pet red squirrels (Deng et al., 2020a) and pet chipmunks (Deng et al., 2018). To our knowledge, this is the first study of *E. bieneusi* in diarrheic pigs in southwest China. This study aimed to determine the molecular prevalence and genotype distribution of *E. bieneusi* in diarrheic pigs in Sichuan and Chongqing Provinces using nested PCR amplicon of the ITS region. The researchers hope that the information from this study will make it possible to take the proper steps to prevent and control *E. bieneusi* infection in pigs and humans by the health authorities.

## Materials and methods

### Collection of fecal samples

Between September 2021 and March 2022, 514 fresh fecal samples were collected from 14 large-scale intensive pig farms in Chongqing (five) and Sichuan (nine) Provinces in southwestern China. All fecal specimens were collected immediately after defecation using sterile disposable latex gloves and placed in individual plastic containers. The date, identification number, and age of each pig were recorded during sampling. The diarrheic pigs in this study were divided into four groups, with one containing 326 suckling pigs (preweaned) aged less than 1 month, 17 weaned piglets aged between 2 and 4 months, 65 fattening pigs aged between 4 and 6 months, and 106 sows aged greater than 6 months (Table 1). At the time of sampling, all pigs were in diarrheic condition. The diarrhea condition ranged from severe watery diarrhea (pink color) to loose diarrhea. The specimens were transported immediately to the laboratory on ice packs and kept in a refrigerator at −20°C until DNA extraction.

**Table 1:** Prevalence of diarrheic pigs by Age groups in Sichuan and Chongqing Provinces (in southwestern China).

Variable	Category	№ positive/№ tested (%)	95% CI	Value of p	Genotypes
Province	Sichuan	326/400 (81.5)	74.3–81.7		PigCE01.01(14), PigCE01.02(6), PigCE01.03(1), PigCE01.04(2), PigCE01.05(2), PigCE01.06(4), PigCE02.01(8), PigCE02.02(1), PigCE02.03(1), PigCE02.04(1), PigCE02.05(1), PigCE03.01(1), PigCE03.02(2), PigCE06.01(6), PigCE06.02(4), PigCE06.03(1), PigCE06.04(1), PigCE06.05(1), PigCE07.01(10), PigCE07.02(7), PigCE07.03(1), PigCE07.04(2), PigCE07.05(7), PigCE07.06(2), PigCE07.07(1), PigCE07.08(1), PigCE07.09(5), PigCE07.10(1), PigCE08.01(3), PigCE08.02(114), PigCE08.03(1), PigCE08.04(2), PigCE08.05(6), PigCE08.06(1), PigCE08.07(23), PigCE08.08(1), PigCE08.09(2), PigCE08.10(4), PigCE08.11(2), PigCE08.12(1), PigCE08.13(2), PigCE08.14(1), PigCE08.15(2), PigCE08.16(3), PigCE08.17(1), PigCE08.18(2), PigCE08.19(1), B (54), BEB17(2) *F* (4)
	Chongqing	84/114 (73.7)	18.3–25.7		PigCE01.01(2), PigCE01.06(2), PigCE01.07(1), PigCE02.06(1), PigCE04.01(1), PigCE05.01(1), PigCE06.01(1), PigCE07.01(1), PigCE07.02(1), PigCE07.04(1), PigCE07.11(1), PigCE07.12(1), PigCE08.02(24), PigCE08.03(1), PigCE08.07(4), PigCE08.15(1), PigCE08.20(1), B (17), CAF1(1), E (6), *F* (13), Peru8(1), CHG7(1),
Age	Suckling piglets(Age ≤ 1 month)	262/326 (80.4)	58.2–66.5		PigCE01.01(8), PigCE01.02(5), PigCE01.03(1), PigCE01.05(1), PigCE02.01(8), PigCE02.02(1), PigCE02.03(1), PigCE02.04(1), PigCE02.05(1), PigCE03.01(1), PigCE03.02(2),PigCE06.01(5), PigCE06.02(4), PigCE06.03(1), PigCE06.04(1), PigCE07.01(8), PigCE07.02(6), PigCE07.03(1), PigCE07.04(1), PigCE07.05(4), PigCE07.06(1), PigCE07.09(4), PigCE07.10(1), PigCE07.12(1), PigCE08.01(3), PigCE08.02(92), PigCE08.03(1), PigCE08.04(2), PigCE08.05(3), PigCE08.06(1), PigCE08.07(20), PigCE08.08(1), PigCE08.09(1), PigCE08.10(4), PigCE08.11(1), PigCE08.12(1), PigCE08.13(1), PigCE08.16(3), PigCE08.17(1), PigCE08.18(2), PigCE08.19(1), B (47), *F* (03), BEB17(02)
	Weaned pigs(1 to 4 months)	11/17 (64.7)	2.7–6.2		PigCE06.01(1), PigCE01.07(1), PigCE08.02(5), B (1), *F* (1), CAF1(1), E (1)
	(4 to 6 months)	47/65 (72.3)	9.9–15.6		PigCE02.06(1), PigCE04.01(1), PigCE05.01(1), PigCE07.02(1), PigCE07.04(1), PigCE07.11(1), PigCE08.02(9), PigCE08.03(1), PigCE08.07(1), PigCE08.15(1), PigCE08.20(1), B (09), CHG7(1), *F* (12), E (5), Peru8(1)
	Sow (≥6 months)	90/106 (84.9)	17.5–23.7	0.138	PigCE01.01(4), PigCE01.02(1), PigCE01.04(2), PigCE01.05(1), PigCE01.06(6), PigCE06.01(1), PigCE06.05(1), PigCE07.01(3), PigCE07.02(1), PigCE07.04(1), PigCE07.05(3), PigCE07.06(1), PigCE07.07(1), PigCE07.08(1), PigCE07.09(1), PigCE08.02(32), PigCE08.05(3), PigCE08.07(6), PigCE08.09(1), PigCE08.11(1), PigCE08.13(1), PigCE08.14(1), PigCE08.15(2), B (14), F (1)
Total		410/514 (79.8)			PigCE01.01(16), PigCE01.02(6), PigCE01.03(1), PigCE01.04(2), PigCE01.05(2), PigCE01.06(6), PigCE01.07(1), PigCE02.01(8), PigCE02.02(1), PigCE02.03(1), PigCE02.04(1), PigCE02.05(1), PigCE02.06(1), PigCE03.01(1), PigCE03.02(2), PigCE04.01(1), PigCE05.01(1), PigCE06.01(7), PigCE06.02(4), PigCE06.03(1), PigCE06.04(1), PigCE06.05(1), PigCE07.01(11), PigCE07.02(8), PigCE07.03(1), PigCE07.04(3), PigCE07.05(7), PigCE07.06(2), PigCE07.07(1), PigCE07.08(1), PigCE07.09(5), PigCE07.10(1), PigCE07.11(1), PigCE07.12(1),PigCE08.01(3), PigCE08.02(138), PigCE08.03(2), PigCE08.04(2), PigCE08.05(6), PigCE08.06(1), PigCE08.07(27), PigCE08.08(1), PigCE08.09(2), PigCE08.10(4), PigCE08.11(2), PigCE08.12(1), PigCE08.13(2), PigCE08.14(1), PigCE08.15(3), PigCE08.16(3), PigCE08.17(1), PigCE08.18(2), PigCE08.19(1), PigCE08.20(1), B (71), BEB17(02), CAF1(1), E (6), *F* (17), Peru8(01), CHG7(1)

### DNA extraction and purification

All the preserved fecal specimens were washed twice with deionized water to remove the preservative or impurities. All fecal specimens of the pigs were sieved (filtered) through an 8.0-cm-diameter sieve with a pore size of 45 μm. The filtrates were concentrated by centrifugation at 15000 *× g* for 10 min. Genomic DNA was extracted from approximately 200 mg (200 μL) from each processed fecal sample using an E.Z.N.A.^®^ Mag- Bind Stool DNA Kit (OMEGA, Biotek Inc. Norcross, GA, United States), according to the manufacturer’s instructions. DNA was eluted in 50 μL of double-deionized distilled water, and DNA quantification was performed using a NanoDrop. Extracted DNA was stored at −20°C until used for PCR analysis.

### PCR amplification

The internal transcribed spacer (ITS) region and sections of the surrounding large and small subunits of the ribosomal RNA gene were amplified using nested PCR to detect the presence of *E. bieneusi* in all the DNA extracted from diarrheic pigs, as previously described by Buckholt et al. (2002). In brief, a final PCR product of approximately 390 bp was produced using the outer primer sets EBITS3 (5′–GGTCATAGGGATGAAGAG–3′) and EBTIS4 (5′–TTCGAGTTCTTTCGCGCTC–3′) and the inner primer sets EBITS1 (5′–GCTCTGAATATCTATGGCT–3′) and EBITS2.4 (5′–ATCGCCGACGGATCCAAGTG–3′). All PCRs were conducted using a 25 μL volume containing 12.5 μL of Taq PCR Master Mix (Sangon Biotech Co., Ltd., Shanghai, China), 1 μL of each primer (0.4 mM), 1 μL of each DNA sample, and 10.5 μL of nuclease-free water. Positive and negative controls were included in all the PCR tests conducted. The PCR cycling conditions for the first PCR consisted of an initial denaturation at 94°C for 3 min, followed by 35 cycles at 94°C for 30 s, 57°C for 30 s, and 72°C for 40 s, with a final extension at 72°C for 10 min. The conditions for the second PCR were the same as those of the first PCR, except for the annealing temperature, which was 55°C for 30 cycles. The secondary PCR products were gel electrophoresed using a 1.5% agarose gel and visualized by the ChemiDoc XRS+ Gel Imaging System (Bio-Rad, California, United States). Workspaces were established for DNA extraction, PCR reagent preparation, and PCR amplification to prevent cross-contamination. Moreover, reagent preparation was performed in a UV-illuminated biosafety cabinet.

### Nucleotide sequencing and analysis

All *E. bieneusi*-positive secondary PCR results were sent for sequencing by Sangon Biotech Co., Ltd. (Shanghai, China). As necessary, sequencing in both directions and sequencing additional PCR products, were utilized to confirm the accuracy of the sequence. The Snap-Gene version 5.1 sequence analysis tool (TechnelysiumPty Ltd., South Brisbane, Australia) was used to view the raw sequencing data in both the forward and reverse directions. Using the Basic Local Alignment Search Tool (BLAST)^1^ and ClustalX 1.83,^2^ the genotypes of *E. bieneusi* were determined by comparing the nucleotide sequences derived from *E. bieneusi* isolates with each other and with published GenBank sequences.

### Phylogenetic and statistical analyses

The phylogenetic tree was built using the Bayesian inference (BI) and Monte Carlo Markov Chain (MCMC) methods in MrBayes version 3.2.6 (Huelsenbeck and Ronquist, 2001). The substitution model that best fit the dataset was assessed using jModelTest 3.07, and GTR + I + G was the best evolutionary model for *E. bieneusi* (Kalyaanamoorthy et al., 2017). After 1,000,000 generations, the posterior probability values were determined. The remaining 75% of the trees produced by BI were used to create a 50% majority rule consensus tree. To assure convergence and previous insensitivity, analyses were repeated three times. The novel ITS sequences of *E. bieneusi* isolates have been deposited in GenBank (accession numbers OP161838 to OP161891). Data were entered using Microsoft Excel, 2010. Data were analyzed using SPSS statistical software (version 20), and the chi-square test (χ^2^) was used to detect significant differences. A *p* value <0.05 was considered statistically significant.

## Results

### *Enterocytozoon bieneusi* prevalence and genotype distribution in diarrheic pigs in Sichuan and Chongqing Provinces of China

A total of 514 fecal samples from diarrheic pigs from 14 farms in the southern Chinese provinces of Sichuan (*n* = 9) and Chongqing (*n* = 5) were analyzed using molecular techniques. In this study, the overall prevalence of *E. bieneusi* in diarrheic pigs was 79.8% (410/514; Table 1). *E. bieneusi* was found in all farms that the pig samples collected from both Sichuan and Chongqing provinces. In Sichuan, the highest (100%) and lowest (60%) prevalences of suckling pigs were found on farms 1 and 7, respectively. In contrast, the prevalences for the sows were highest (100%) and lowest (63.6%) on farms 2 and 8 and farm 7, respectively. In Chongqing, 100% (14/14) and 0% (0/1) were recorded as the highest and lowest prevalences in sows from farms 10 and 12, respectively. In fattening pigs, 21 of 25 (83.3%) and 11 of 25 (55%) were recorded as the highest and lowest prevalences on farms 13 and 11, respectively. In suckling pigs, 5 of 6 (83.3%) and 2 of 5 (40%) were the highest and lowest prevalences recorded on farms 13 and 12, respectively. Based on nucleotide sequence analysis of the ITS region, all farms had at least two genotypes except farm 14, which had one genotype. Moreover, the genotype PigCE08.02 was the most common and was found on all farms except farm 4 (Table 2).

**Table 2:** *E. bieneusi* prevalence and genotypes identified in diarrheic pigs from different farms of Sichuan and Chongqing Provinces

Province	Farms	Age group	№ positive/№ tested (%)	Genotypes (Number)
Sichuan	1	Suckling	23/23 (100.0)	PigCE01.01(1), PigCE02.01(1), PigCE02.02(1), PigCE02.03(1), PigCE06.01(1), PigCE06.01(1), PigCE07.01(2), PigCE07.02(3), PigCE07.03(1), PigCE08.01(1), PigCE08.02(3), PigCE08.03(1), PigCE08.04(1), PigCE08.08(1), B (4),
	2	Suckling	45/48 (93.8)	PigCE01.01(6), PigCE01.02(3), PigCE01.03(1), PigCE01.04(1), PigCE02.01(4), PigCE06.01(2), PigCE06.03(1), PigCE06.04(1), PigCE07.01(3), PigCE07.02(3), PigCE07.04(1), PigCE07.05(2), PigCE07.06(1), PigCE08.02(6), PigCE08.05(2), PigCE08.06(1), PigCE08.07(3), PigCE08.16(1), B (4),
		Sow	5/5 (100.0)	PigCE01(1), PigCE07.05(1), PigCE07.07(1), PigCE08.02(1), PigCE08.05(1)
	3	Suckling	39/50 (78.0)	B (3), PigCE02.04(1), PigCE03.01(1), PigCE03.02(2), PigCE06.01(2), PigCE06.02(1), PigCE07.05(1), PigCE07.05(1), PigCE07.10(1), PigCE08.02(13), PigCE08.07(8), PigCE08.09(1), PigCE08.10(3), PigCE08.11(1), PigCE08.12(1),
	4	Sow	46/50 (92.0)	PigCE01.01(2), PigCE01.02(1), PigCE01.041(1), PigCE01.05(1), PigCE01.06(3), PigCE06.01(1), PigCE06.05(1), PigCE07.01(1), PigCE07.05(2), PigCE07.08(1), PigCE07.09(1), PigCE08.01(17), PigCE08.05(2), PigCE08.07(2), PigCE08.09(1), PigCE08.11(1), PigCE08.13(1), PigCE08.14(1), PigCE08.15(2), B (3), F (1)
	5	Suckling	22/23 (95.7)	PigCE01.02(1), PigCE02.01(1), PigCE07.01(1), PigCE07.09(3), PigCE08.01(1), PigCE08.02(7), PigCE08.04(1), PigCE08.07(2), PigCE08.16(1), PigCE08.17(1), B (3),
	6	Suckling	28/30 (93.3)	PigCE01.01(4), PigCE01.02(1), PigCE07.01(1), PigCE07.05(1), PigCE07.09(1), PigCE08.01(1), PigCE08.02(3), PigCE08.07(2), PigCE08.16(1), PigCE08.18(2), B (10), F (1)
		Sow	13/20 (65.0)	PigCE01.06(1), PigCE07.01(1), PigCE07.06(1), PigCE08.02(5), B (5),
	7	Suckling	24/40 (60.0)	PigCE08.02(13), PigCE08.08(1), PigCE08.19(1), B (9)
		Sow	7/11 (63.6)	PigCE07.02(1), PigCE07.04(1), PigCE08.02(4), B (1),
	8	Suckling	34/45 (75.6)	PigCE06.01(1), PigCE08.02(20), PigCE08.05(1), PigCE08.07(2), PigCE08.13(1), B (5), BEB17(2), *F* (2)
		Sow	5/5 (100.0)	PigCE08.02(4), PigCE08.07(1)
	9	Suckling	35/50 (70.0)	PigCE01.01(1), PigCE01.05(1), PigCE02.02(2), PigCE02.05(1), PigCE06.01(1), PigCE07.01(1), PigCE08.02(18), PigCE08.07(2), PigCE08.10(1), B (7),
Chongqing	10	Fattening	15/20 (75)	PigCE02.06 (1), PigCE07.04(01), PigCE08.02(5), PigCE08.03(1), PigCE08.15(1), B (6)
		Sow	14/14 (100)	PigCE01.01(2), PigCE01.06(2), PigCE07.01(1), PigCE08.02(1), PigCE08.07(3), B (5)
	11	Fattening	11/20 (55.0)	PigCE07.01(1), PigCE07.11(1), PigCE08.02(4), PigCE08.07(1), PigCE08.20(1), B (3)
	12	Weaned	05/9 (55.6)	PigCE01.07(1), PigCE08.02(2), CAF1(1), E (1)
		Suckling	05/6 (83.3)	PigCE08.02(5)
		Sow	0/1 (0.0)	
	13	Fattening	21/25 (84.0)	PigCE04.01(1), PigCE05.01(1) E (5), CHG7 (1), F (12), Peru8 (1)
		Weaned	6/8 (75)	PigCE06.01(1), PigCE08.02(3), B (1), F (1),
		Suckling	5/6 (83.3)	PigCE07.12(1), PigCE08.02(2), B (2)
	14	Suckling	2/5 (40.0)	PigCE08.02(2)

The prevalences of *E. bieneusi* in the fecal samples of the diarrheic pigs in Sichuan and Chongqing were 81.5% (326/400) and 73.7% (84/114), respectively (Table 1). Pigs that tested positive for *E. bieneusi* were discovered on all the farms sampled, and the prevalences of *E. bieneusi* ranged from 40 to 100% among the 14 farms included in this study. The prevalence of *E. bieneusi* was highest (84.9%) in the sow age group, and lowest in weaned pigs (64.7%; Table 1). However, this difference in the prevalence of *E. bieneusi* among the different age groups was not statistically significant (χ2 = 6.437, df = 3, *p* > 0.05).

**Figure 1:**
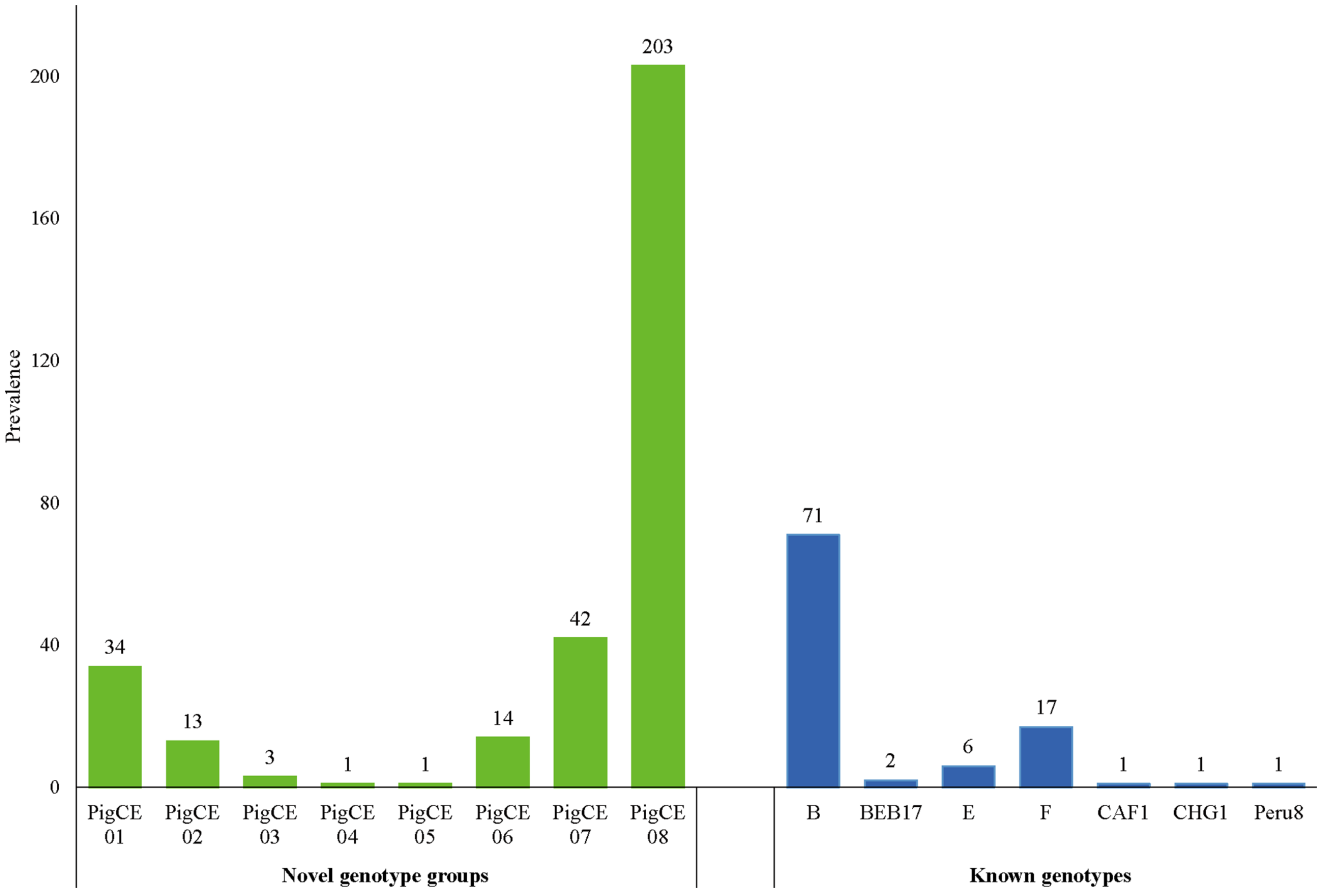
Distribution of the eight novel genotype groups and seven known genotypes.

Based on the ITS analysis, 61 different genotypes were identified, which consisted of seven known genotypes (E, F, CHG7, Peru8, CAF1, B, and BEB17) and 54 novel genotypes as variants of eight known genotypes (SDD2, A, B, HLJD-IV, PigSpEb1, O, JLD-I, and BEB17). These 54 novel genotypes were classified into eight genotype groups PigCE01(7), PigCE02(6), PigCE03(2), PigCE04(1), PigCE05(1), PigCE06(5), PigCE07(12), and PigCE08(20) based on their sequence similarities (Figure 1).

### *Enterocytozoon bieneusi* genotypes in diarrheic pigs

A sequencing study of 410 pig-derived *E. bieneusi* isolates revealed 23 polymorphism locations within the ITS regions and 243 base pairs (Supplementary Figure S4). Homology analysis of *E. bieneusi* ITS sequences indicated the presence of 61 different genotypes, including seven known genotypes: B (*n* = 77), F (*n* = 17), E (*n* = 6), BEB17 (*n* = 2), CAF1 (*n* = 1), CHG7 (*n* = 1) and Peru8 (*n* = 1) and 54 different and novel genotypes categorized under PigCE01 to PigCE08 (Accession no. OP161838 to OP161891) containing one to five single-nucleotide polymorphisms (SNPs). Thus, the genotypes, PigCE01 to PigCE08, have a total of 54 single SNPs (Supplementary Figure S1). Among the known genotypes, genotype B was the most dominant (17.3%, 71/410) and was 100% identical to the previously reported (GenBank No. AF101198, isolated from *Homo sapiens*). This was followed by genotype F (4.2%, 17/410) and genotype E (1.5%, 6/410), which were 100% identical to the sequences AF135833 and AF135832 isolated from pigs, respectively.

Genotype group PigCE08 was the most prevalent (49.5%, 203/410) of the novel genotype groups discovered in this study and was found to be present in 172 and 31 diarrheic pigs from Sichuan and Chongqing, respectively. Only Sichuan samples were discovered to contain the PigCE03 genotype. Genotypes PigCE04 and PigCE05 were found in Chongqing samples, each with one isolate. Genotypes PigCE01, PigCE02, PigCE06, PigCE07, and PigCE08 were found in both Sichuan and Chongqing province samples. Genotype group PigCE07 (10.2%, 42/410) was the second most common genotype among the novel genotype groups. Followed by genotype group PigCE01 (8.3%, 34/410), genotype group PigCE06 (3.2%, 13/410) and genotype group PigCE03 (0.7%, 3/410). The remaining genotype groups, PigCE04 and PigCE05, each had one isolate (0.2%, 1/410).

The ITS sequences of the 54 novel genotypes were highly similar to those of the previously described genotypes BEB17, B, SDD2, A, JLD-I, HLJD-IV, O, and PigSpEb1 (Rinder et al., 1997; Da Silva Fiuza et al., 2016; Dashti et al., 2020; Supplementary Figure S1). Compared to genotypes A (accession No. AF101197) and BEB17(accession No. KT984495), both the novel *E. bieneusi* genotype groups PigCE02 and PigCE08 were discovered to contain a range of one to five SNPs. Genotype groups PigCE01 and PigCE06 compared to their reference genotypes SDD2 (accession No. MN704922) and JLD-I (accession No. KX383625), distinguished by two to five nucleotide positions, respectively. Genotype group PigCE07 showed a range of one to five SNPs in contrast to genotype B (accession No. AF101198). PigCE03 diverged from genotype HLJD-IV (accession No. KX383621) at four and five nucleotide positions. One nucleotide location distinguished genotype group PigCE04 from genotype O (accession No. AF267145). Genotype group PigCE05 diverged by five nucleotides from PigSpEb1(accession No. MN699291).

### *Enterocytozoon bieneusi* phylogenetic relationship

**Figure 2:**
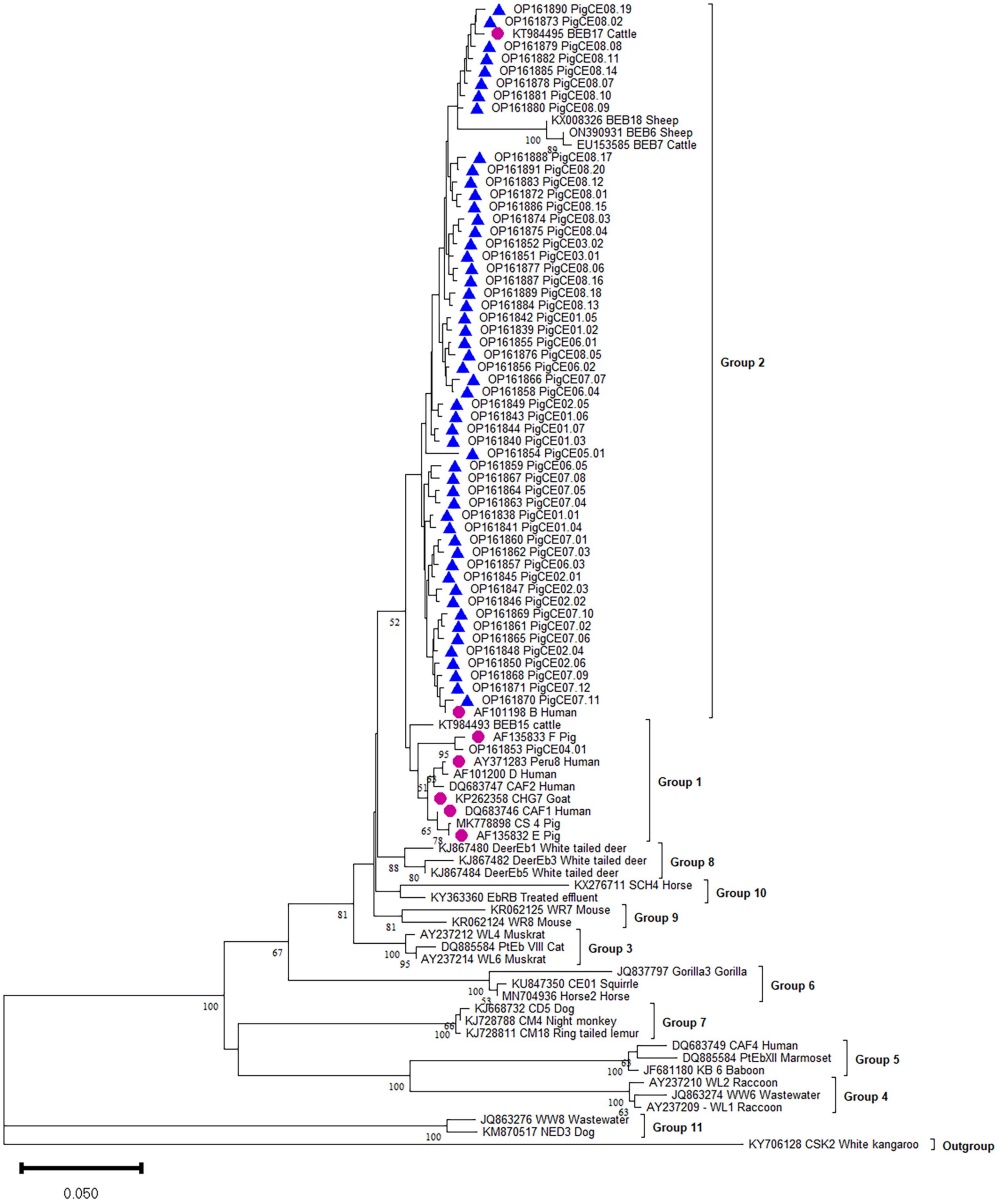
Phylogenetic analysis of the ITS of rRNA of *E. bieneusi* genotype identified in the present study and representative reported genotypes. The phylogenetic tree was constructed in Bayesian interference (BI) methods. Statistically significant posterior frequencies (50 and higher) are indicated on the branches. Known and novel *E. bieneusi* ITS genotypes identified in this study are shown with purple circles and blue triangles, respectively.

The phylogenetic analysis showed that the six known (B, CAF1, CHG7, E, F, and Peru8) genotypes were assigned to zoonotic potential Group 1. Group 2 consists of one known genotype (BEB17) and all 54 novel genotypes (Figure 2).

## Discussion

According to the findings of this research, the overall prevalence of *E. bieneusi* infection in diarrheic pigs in the 14 farms in the provinces of Sichuan and Chongqing was 79.8%. This high prevalence of *E. bieneusi* in Chongqing and Sichuan is not surprising because all the samples used in this study showed clinical diarrhea symptoms. Our results were higher than those of other previous studies conducted in other provinces of China in asymptomatic pigs, such as Jilin (60.3%, 94/156; Li et al., 2014b), Xinjiang (48.6%, 389/801; Li et al., 2019b), Hainan (46.8%, 88/188; Zhou et al., 2020), Heilongjiang (45.3%, 39/86; Li et al., 2014a), Henan (45.5%, 408/897; Wang et al., 2018a), Tibet (43.2%, 309/715; Li et al., 2019a), northeast China (52.1%, 456/875; Wan et al., 2016), southern China (Zhejiang, Guangdong, and Yunnan provinces; 31.57%, 125/396; Zou et al., 2018), southwest China (Kangding, Yaan, Qionglai; 31.2%, 83/266; Luo et al., 2019) and Fujian (24.4%, 177/725; Zhang et al., 2020). However, the prevalence was lower than that in Heilongjiang Province (83.2%, 79/95; Zhao et al., 2014) and similar to that in Shaanxi Province, northwestern China (78.9%, 325/560; Wang et al., 2018b; Supplementary Figure S2). In addition, in symptomatic pigs, our findings were higher than those of the studies carried out in Germany (66.7%, 4/6; Rinder et al., 2000) and two studies from northeast China (45.1%, 51/113; 35.1%, 61/174; Li et al., 2014a; Wan et al., 2016). The majority of the studies carried out to date were in asymptomatic pigs, with the exception of a few studies (Rinder et al., 2000; Li et al., 2014a,c; Valenčáková and Danišová, 2019).

*E. bieneusi* is one of the most clinically identified intestinal parasites in humans and animals (Zhao et al., 2014). Our results indicated that the prevalence of *E. bieneusi* varied among the age groups, with the highest prevalence in sow (84.9%) and the lowest prevalence in weaned (64.7%); however, the difference was not statistically significant (χ2 = 6.437, df = 3, *p* = 0.092). Our findings were higher than those in previous studies conducted in the Czech Republic (80%; Sak et al., 2008), and China, including Shaanxi (47.4%, 27/57; Wang et al., 2018b), Henan (33.9%, 73/215; Wang et al., 2018a), southern China (22.88%, 54/236; Zou et al., 2018), and Xinjiang (11.3%, 25/222; Li et al., 2019b). The high prevalence in this age range may be mainly attributed to inadequate sanitation in environments with high pig numbers, increasing the likelihood of *E. bieneusi* infection transfer between individuals (Sak et al., 2008). Moreover, a study conducted on both diarrheic and nondiarrheic piglets by Jeong et al. (2007) found that diarrheic piglets had a higher rate of infection with *E.bieneusi* (16%, 38/237) than healthy piglets (12%, 29/235). Additionally, Li et al. (2014a) confirmed a high prevalence (45.1%, 51/113) of *E. bieneusi* in symptomatic pigs in northeast China. However, it is unknown whether life-threatening diarrhea in the animals used in the study was related to the virulence of *E. bieneusi* (Li et al., 2014a). Unlike the current study, two studies performed on diarrheic pigs in northeast China showed that weaned pigs had the highest rates of infection (35%, 7/20; 63.6%, 21/33) of *E. bieneusi* compared to preweaned pigs (suckling pigs; 3.6%, 1/28; 41.0%, 25/61; Li et al., 2014a,c). The high prevalence of *E. bieneusi* in diarrheic pigs indicates that *E. bieneusi* could be a significant pathogen that causes diarrhea in different domestic and wild mammals and immunocompromised humans (Matos et al., 2012; Li et al., 2014a). However, further study is required to confirm whether *E. bieneusi* is a life-threatening diarrhea causative agent alone or behaves as a co-infected pathogen with other enteropathogens. This result suggests that *E. bieneusi* is a potential cause of diarrhea in all age groups.

The high prevalence of *E. bieneusi* on all farms among diarrheic pigs in Sichuan and Chongqing Provinces suggests that the pathogen was prevalent throughout the area. This harm may negatively impact productivity and provide a considerable danger of zoonotic disease transmission, particularly among immunocompromised individuals. Therefore, farmers, veterinarians, and public health authorities should take serious steps to reduce the infection rates within the farms and the provinces. In addition, farmers should take precautions not to be infected by their animals, as pigs serve as one of the potential host reservoirs for *E. bieneusi* (Ruviniyia et al., 2020).

Currently, the ITS locus is the standard method for determining the genotype of *E. bieneusi* because the species has considerable genetic diversity. This investigation revealed various *E. bieneusi* genotypes in the studied areas. The ITS sequences of 410 *E. bieneusi* isolates showed seven known types (B, F, E, BEB17, CAF1, CHG7, and Peru8) and 54 novel types (PigCE01 to 08). These 54 new genotypes are variants of eight known genotypes (SDD2, A, B, HLJD-IV, PigSpEb1, O, JLD-I, and BEB17) based on their sequence similarities. Moreover, the 54 novel genotypes differed from each other by one to five SNPs. In this study, genotypes B and F were the most commonly identified genotypes. Moreover, human microsporidiosis has also been linked to genotypes B and F in some parts of the world. Genotype B, for example, has been found in humans in Germany (Rinder et al., 1997). Genotype F was also reported to be first isolated from pigs in Germany (Rinder et al., 2000). To date, in pigs, at least 139 *E. bieneusi* genotypes have been identified (e.g., BEB4, CS-4, D, EbpA, EbpB, EbpC, EbpD, EbpE, G, H, Henan-IV, O, PigEBITS1-8, etc.). Most of these belong to human-pathogenic Group 1, which supports the likelihood of zoonotic transmission between pigs and humans (Wang et al., 2018a; Li et al., 2019d, 2020). Genotypes B, E, F, CHG7, Peru8, and CAF1 of the present study also clustered in Group 1. In addition, genotypes E, F, and CHG7 were also isolated from sheep, goats, cattle, and wild boars. Therefore, to prevent the transmission of *E. bieneusi* from pigs to humans, the close contact between *E. bieneusi*-carrying pigs and vulnerable individuals must be reduced. All the novel genotypes and known BEB17 genotypes were clustered in Group 2, indicating that Group 2 is a potential reservoir for zoonotic diseases (Liu et al., 2021).

The present study found no significant correlations between prevalence and age groups. Therefore, future studies should include more samples and variables (such as sexes, seasons, husbandry management, and rearing patterns) to document the infection condition of *E. bieneusi* in pigs and provide enough practical suggestions to prevent infection in pigs, animals, and humans. These results are expected to increase our understanding of the distribution of *E. bieneusi* genotypes throughout the provinces and have implications for managing *E. bieneusi* infections in animals and people.

## Conclusion

To the best of our knowledge, this is the first report of *E. bieneusi* in diarrheic pigs in Sichuan and Chongqing Provinces, China. The overall prevalence of *E. bieneusi* was 79.8%, and all farm and age categories of pigs were found to be infected with *E. bieneusi*. Sixty-one genotypes, including seven known and 54 novel genotypes (classified into eight groups), were identified, with the *E. bieneusi* belonging to zoonotic Groups 1 and 2. These findings indicate that diarrheic pigs could be a potential reservoir host, that could contaminate the environment and be a source of microsporidia infections in humans and other animals.

## Data availability statement

The datasets presented in this study can be found in online repositories. The names of the repository/repositories and accession number(s) can be found in the article/Supplementary material.

## Ethics statement

This research was reviewed and approved by the Institutional Animal Care and Use Committee (IACUC) of Southwest University (approval no. IACUC-20220420-04). All fecal samples were collected based on the accessibility of the animals for sampling and the owner’s or farm manager’s willingness to participate in the study.

## Author contributions

GP, SG, ZZ, JB, and MA-K conceived and designed the experiments. YY and XL collect the specimens. JW, SG, QH, and LL prepared the materials and performed the experiment. SG, HX, and TL analyzed the data. SG, GP, XM, and JC wrote the manuscript. All authors contributed to the article and approved the submitted version.

## Funding

This work was supported by the Natural Science Foundation of Chongqing, China (nos. cstc2019yszx-jcyjX0010, cstc2021jcyj-msxmX1003, and cstc2021jcyj-cxttX0005), and the opening fund of Chongqing Key Laboratory of Microsporidia Infection and Control (no. CKMIC202101).

## Conflict of interest

The authors declare that the research was conducted in the absence of any commercial or financial relationships that could be construed as a potential conflict of interest.

## Publisher’s note

All claims expressed in this article are solely those of the authors and do not necessarily represent those of their affiliated organizations, or those of the publisher, the editors and the reviewers. Any product that may be evaluated in this article, or claim that may be made by its manufacturer, is not guaranteed or endorsed by the publisher.

## Footnotes

^1^ http://blast.ncbi.nlm.nih.gov/Blast.cgi

^2^ http://www.clustal.org/

## References

[ref1] Amer S. Kim S. Han J. I. Na K. J. (2019). Prevalence and genotypes of *Enterocytozoon bieneusi* in wildlife in Korea: a public health concern. Parasit. Vectors 12:160. doi: 10.1186/S13071-019-3427-6, PMID: 30961667 PMC6454782

[ref2] Buckholt M. A. Lee J. H. Tzipori S. (2002). Prevalence of Enterocytozoon bieneusi in swine: an 18-month survey at a slaughterhouse in Massachusetts. Appl. Environ. Microbiol. 68, 2595–2599. doi: 10.1128/Aem.68.5.2595-2599.2002, PMID: 11976142 PMC127518

[ref3] Da Silva Fiuza V. R. Lopes C. W. De Oliveira F. C. Fayer R. Santin M. (2016). New findings of *Enterocytozoon bieneusi* in beef and dairy cattle in Brazil. Vet. Parasitol. 216, 46–51. doi: 10.1016/J.Vetpar.2015.12.008, PMID: 26801594

[ref4] Dashti A. Rivero-Juárez A. Santín M. George N. S. Köster P. C. López-López P.. (2022). Diarrhoea-causing enteric Protist species in intensively and extensively raised pigs (*Sus scrofa domesticus*) in southern Spain. Part I: prevalence and genetic diversity. Transbound. Emerg. Dis. 69, E1051–E1064. doi: 10.1111/tbed.14388, PMID: 34755463

[ref5] Dashti A. Rivero-Juarez A. Santín M. López-López P. Caballero-Gómez J. Frías-Casas M.. (2020). *Enterocytozoon bieneusi* (microsporidia): identification of novel genotypes and evidence of transmission between sympatric wild boars (*Sus scrofa ferus*) and Iberian pigs (*Sus scrofa domesticus*) in southern Spain. Transbound. Emerg. Dis. 67, 2869–2880. doi: 10.1111/Tbed.13658, PMID: 32500974

[ref6] Deng L. Chai Y. Luo R. Yang L. Yao J. Zhong Z.. (2020a). Occurrence and genetic characteristics of *Cryptosporidium*. and Enterocytozoon bieneusi in pet red squirrels (*Sciurus vulgaris*) in China. Sci. Rep. 10, 1–10. doi: 10.1038/s41598-020-57896-w 31974403 PMC6978461

[ref7] Deng L. Chai Y. Xiang L. Wang W. Zhou Z. Liu H.. (2020b). First identification and genotyping of *Enterocytozoon bieneusi* and *Encephalitozoon* spp. in pet rabbits in China. BMC Vet. Res. 16, 1–8. doi: 10.1186/s12917-020-02434-z 32571322 PMC7310219

[ref8] Deng L. Li W. Zhong Z. Chai Y. Yang L. Zheng H.. (2018). Molecular characterization and new genotypes of *Enterocytozoon bieneusi* in pet chipmunks (*Eutamias asiaticus*) in Sichuan Province. China. Bmc Microbiol 18:37. doi: 10.1186/S12866-018-1175-Y, PMID: 29669519 PMC5907217

[ref9] Deng L. Yue C. J. Chai Y. J. Wang W. Y. Su X. Y. Zhou Z. Y.. (2019). New genotypes and molecular characterization of *Enterocytozoon bieneusi* in pet birds in southwestern China. Int J Parasitol Parasites Wildl 10, 164–169. doi: 10.1016/J.Ijppaw.2019.08.001, PMID: 31667078 PMC6811997

[ref10] Didier E. S. Weiss L. M. (2011). Microsporidiosis: not just in Aids patients. Curr. Opin. Infect. Dis. 24, 490–495. doi: 10.1097/Qco.0b013e32834aa152, PMID: 21844802 PMC3416021

[ref11] Dubreuil J. D. Isaacson R. E. Schifferli D. M. (2016). Animal Enterotoxigenic *Escherichia coli*. EcoSal Plus 7:2016. doi: 10.1128/Ecosalplus.Esp-0006-2016, PMID: 27735786 PMC5123703

[ref12] Gyles C. L. Fairbrother J. M. (2010). “*Escherichia coli*” in Pathogenesis of bacterial infections in animals. eds. Gyles C. L. Prescott J. F. Glenn Songer J. Thoen C. O.. 4th Edn. (New York, Ny: Wiley-Blackwell)

[ref13] Huelsenbeck J. P. Ronquist F. (2001). Mrbayes: Bayesian inference of phylogenetic trees. Bioinformatics 17, 754–755. doi: 10.1093/Bioinformatics/17.8.754 11524383

[ref14] Jeong D. K. Won G. Y. Park B. K. Hur J. You J. Y. Kang S. J.. (2007). Occurrence and genotypic characteristics of *Enterocytozoon bieneusi* in pigs with diarrhea. Parasitol. Res. 102, 123–128. doi: 10.1007/S00436-007-0740-3, PMID: 17874327

[ref15] Kalyaanamoorthy S. Minh B. Q. Wong T. K. F. Von Haeseler A. Jermiin L. S. (2017). Modelfinder: fast model selection for accurate phylogenetic estimates. Nat. Methods 14, 587–589. doi: 10.1038/Nmeth.4285, PMID: 28481363 PMC5453245

[ref16] Karim M. R. Wang R. Dong H. Zhang L. Li J. Zhang S.. (2014). Genetic polymorphism and zoonotic potential of *Enterocytozoon bieneusi* from nonhuman primates in China. Appl. Environ. Microbiol. 80, 1893–1898. doi: 10.1128/Aem.03845-13, PMID: 24413605 PMC3957649

[ref18] Li W. Deng L. Wu K. Huang X. Song Y. Su H.. (2017). Presence of zoonotic *Cryptosporidium scrofarum*, *Giardia duodenalis* assemblage a and *Enterocytozoon bieneusi* genotypes in captive Eurasian wild boars (*Sus scrofa*) in China: potential for zoonotic transmission. Parasit. Vectors 10:10. doi: 10.1186/S13071-016-1942-2, PMID: 28061911 PMC5219718

[ref19] Li W. Diao R. Yang J. Xiao L. Lu Y. Li Y.. (2014a). High diversity of human-pathogenic *Enterocytozoon bieneusi* genotypes in swine in Northeast China. Parasitol. Res. 113, 1147–1153. doi: 10.1007/S00436-014-3752-9, PMID: 24442159

[ref20] Li W. Feng Y. Santin M. (2019c). Host specificity of *Enterocytozoon bieneusi* and public health implications. Trends Parasitol. 35, 436–451. doi: 10.1016/J.Pt.2019.04.004, PMID: 31076351

[ref21] Li W. Feng Y. Xiao L. (2020). Diagnosis and molecular typing of *Enterocytozoon bieneusi*: the significant role of domestic animals in transmission of human Microsporidiosis. Res. Vet. Sci. 133, 251–261. doi: 10.1016/J.Rvsc.2020.09.030, PMID: 33035931

[ref22] Li W. Feng Y. Zhang L. Xiao L. (2019d). Potential impacts of host specificity on zoonotic or interspecies transmission of *Enterocytozoon bieneusi*. Infect. Genet. Evol. 75:104033. doi: 10.1016/J.Meegid.2019.104033, PMID: 31494271

[ref23] Li W. Li Y. Li W. Yang J. Song M. Diao R.. (2014b). Genotypes of *Enterocytozoon bieneusi* in livestock in China: high prevalence and zoonotic potential. PLoS One 9:E97623. doi: 10.1371/Journal.Pone.0097623, PMID: 24845247 PMC4028308

[ref24] Li W. Tao W. Jiang Y. Diao R. Yang J. Xiao L. (2014c). Genotypic distribution and phylogenetic characterization of *Enterocytozoon bieneusi* in diarrheic chickens and pigs in multiple cities, China: potential zoonotic transmission. PLoS One 9:E108279. doi: 10.1371/Journal.Pone.0108279, PMID: 25255117 PMC4177920

[ref25] Li D. F. Zhang Y. Jiang Y. X. Xing J. M. Tao D. Y. Zhao A. Y.. (2019b). Genotyping and zoonotic potential of *Enterocytozoon bieneusi* in pigs in Xinjiang, China. Front. Microbiol. 10:2401. doi: 10.3389/Fmicb.2019.02401, PMID: 31695688 PMC6817468

[ref26] Li D. Zheng S. Zhou C. Karim M. R. Wang L. Wang H.. (2019a). Multilocus typing of *Enterocytozoon bieneusi* in pig reveals the high prevalence, zoonotic potential, host adaptation and geographical segregation in China. J. Eukaryot. Microbiol. 66, 707–718. doi: 10.1111/Jeu.12715, PMID: 30723969

[ref27] Liu H. Ni H. Xu J. Wang R. Li Y. Shen Y.. (2021). Genotyping and zoonotic potential of *Cryptosporidium* and *Enterocytozoon bieneusi* in pigs transported across regions in China. Microb. Pathog. 154:104823. doi: 10.1016/J.Micpath.2021.104823, PMID: 33689811

[ref28] Luo R. Xiang L. Liu H. Zhong Z. Liu L. Deng L.. (2019). First report and multilocus genotyping of *Enterocytozoon bieneusi* from Tibetan pigs in southwestern China. Parasite 26:24. doi: 10.1051/Parasite/2019021, PMID: 31041895 PMC6492536

[ref29] Matos O. Lobo M. L. Xiao L. (2012). Epidemiology of *Enterocytozoon bieneusi* infection in humans. J. Parasitol. Res. 2012:981424. doi: 10.1155/2012/981424, PMID: 23091702 PMC3469256

[ref31] Rinder H. Katzwinkel-Wladarsch S. Löscher T. (1997). Evidence for the existence of genetically distinct strains of *Enterocytozoon bieneusi*. Parasitol. Res. 83, 670–672. doi: 10.1007/S004360050317, PMID: 9272556

[ref32] Rinder H. Thomschke A. Dengjel B. Gothe R. Löscher T. Zahler M. (2000). Close genotypic relationship between *Enterocytozoon bieneusi* from humans and pigs and first detection in cattle. J. Parasitol. 86, 185–188. doi: 10.1645/0022-3395(2000)086[0185:Cgrbeb]2.0.Co;2, PMID: 10701590

[ref33] Ruviniyia K. Abdullah D. A. Sumita S. Lim Y. A. L. Ooi P. T. Sharma R. S. K. (2020). Molecular detection of porcine *Enterocytozoon bieneusi* infection in peninsular Malaysia and epidemiological risk factors associated with potentially zoonotic genotypes. Parasitol. Res. 119, 1663–1674. doi: 10.1007/S00436-020-06648-W, PMID: 32219552

[ref34] Sak B. Kvác M. Hanzlíková D. Cama V. (2008). First report of *Enterocytozoon bieneusi* infection on a pig farm in the Czech Republic. Vet. Parasitol. 153, 220–224. doi: 10.1016/J.Vetpar.2008.01.043, PMID: 18342450

[ref35] Santín M. Fayer R. (2011). Microsporidiosis: *Enterocytozoon bieneusi* in domesticated and wild animals. Res. Vet. Sci. 90, 363–371. doi: 10.1016/J.Rvsc.2010.07.014, PMID: 20699192

[ref36] Stentiford G. D. Becnel J. Weiss L. M. Keeling P. J. Didier E. S. Williams B. P.. (2016). Microsporidia – emergent pathogens in the global food chain. Trends Parasitol. 32, 336–348. doi: 10.1016/J.Pt.2015.12.004, PMID: 26796229 PMC4818719

[ref37] Valenčáková A. Danišová O. (2019). Molecular characterization of new genotypes *Enterocytozoon bieneusi* in Slovakia. Acta Trop. 191, 217–220. doi: 10.1016/J.Actatropica.2018.12.031, PMID: 30586572

[ref38] Wan Q. Lin Y. Mao Y. Yang Y. Li Q. Zhang S.. (2016). High prevalence and widespread distribution of zoonotic *Enterocytozoon bieneusi* genotypes in swine in Northeast China: implications for public health. J. Eukaryot. Microbiol. 63, 162–170. doi: 10.1111/Jeu.12264, PMID: 26333563

[ref39] Wang S. S. Li J. Q. Li Y. H. Wang X. W. Fan X. C. Liu X.. (2018b). Novel genotypes and multilocus genotypes of *Enterocytozoon bieneusi* in pigs in northwestern China: a public health concern. Infect. Genet. Evol. 63, 89–94. doi: 10.1016/J.Meegid.2018.05.015, PMID: 29792989

[ref40] Wang S. S. Wang R. J. Fan X. C. Liu T. L. Zhang L. X. Zhao G. H. (2018c). Prevalence and genotypes of *Enterocytozoon bieneusi* in China. Acta Trop. 183, 142–152. doi: 10.1016/J.Actatropica.2018.04.017 29660311

[ref41] Wang H. Zhang Y. Wu Y. Li J. Qi M. Li T.. (2018a). Occurrence, molecular characterization, and assessment of zoonotic risk of *Cryptosporidium* spp., *Giardia duodenalis*, and *Enterocytozoon bieneusi* in pigs in Henan, Central China. J. Eukaryot Microbiol. 65, 893–901. doi: 10.1111/Jeu.12634, PMID: 29752883

[ref42] Yu Z. Wen X. Huang X. Yang R. Guo Y. Feng Y.. (2020). Molecular characterization and zoonotic potential of *Enterocytozoon bieneusi*, *giardia Duodenalis* and *Cryptosporidium* spp. in farmed masked palm civets (*Paguma larvata*) in southern China. Parasit. Vectors. 13:403. doi: 10.1186/S13071-020-04274-0, PMID: 32771043 PMC7414269

[ref43] Zhang N. Wu R. Ji T. Cui L. L. Cao H. X. Li D.. (2020). Molecular detection, multilocus genotyping, and population genetics of *Enterocytozoon bieneusi* in pigs in southeastern China. J. Eukaryot. Microbiol. 67, 107–114. doi: 10.1111/Jeu.12759, PMID: 31486160

[ref44] Zhang Y. Xie J. Mi R. Ling H. Luo L. Jia H.. (2021). Molecular detection and genetic characterization of *Toxoplasma gondii* in pork from Chongqing, Southwest China. Acta Trop. 224:106134. doi: 10.1016/J.Actatropica.2021.106134, PMID: 34509456

[ref45] Zhao W. Zhang W. Yang F. Cao J. Liu H. Yang D.. (2014). High prevalence of *Enterocytozoon bieneusi* in asymptomatic pigs and assessment of zoonotic risk at the genotype level. Appl. Environ. Microbiol. 80, 3699–3707. doi: 10.1128/Aem.00807-14, PMID: 24727270 PMC4054152

[ref46] Zhou H. H. Zheng X. L. Ma T. M. Qi M. Zhou J. G. Liu H. J.. (2020). Molecular detection of *Enterocytozoon bieneusi* in farm-raised pigs in Hainan Province, China: infection rates, genotype distributions. And Zoonotic Potential. Parasite 27:12. doi: 10.1051/Parasite/2020009, PMID: 32129760 PMC7055476

[ref47] Zou Y. Hou J. L. Li F. C. Zou F. C. Lin R. Q. Ma J. G.. (2018). Prevalence and genotypes of *Enterocytozoon bieneusi* in pigs in southern China. Infect. Genet. Evol. 66, 52–56. doi: 10.1016/J.Meegid.2018.09.006, PMID: 30218706

[ref48] Zou Y. Zheng W. B. Song H. Y. Xia C. Y. Shi B. Liu J. Z.. (2019). Prevalence and genetic characterization of *Enterocytozoon bieneusi* and *Giardia duodenalis* in Tibetan pigs in Tibet, China. Infect. Genet. Evol. 75:104019. doi: 10.1016/J.Meegid.2019.104019, PMID: 31470093

