# Supplementary material for: Prevalence and genotyping distribution of Enterocytozoon bieneusi in diarrheic pigs in Chongqing and Sichuan provinces, China
Source: Front Microbiol. 2022 Oct 13;13:1025613. doi: 10.3389/fmicb.2022.1025613 (PMC9608567; doi:10.3389/fmicb.2022.1025613)
Supplement: Supplementary file 1 [file Data_Sheet_1.PDF]

Supplementary Material

1.1 Supplementary Figures

Supplementary Figure 1. Multiple alignment of the 54 novel genotypes based on their Eight groups

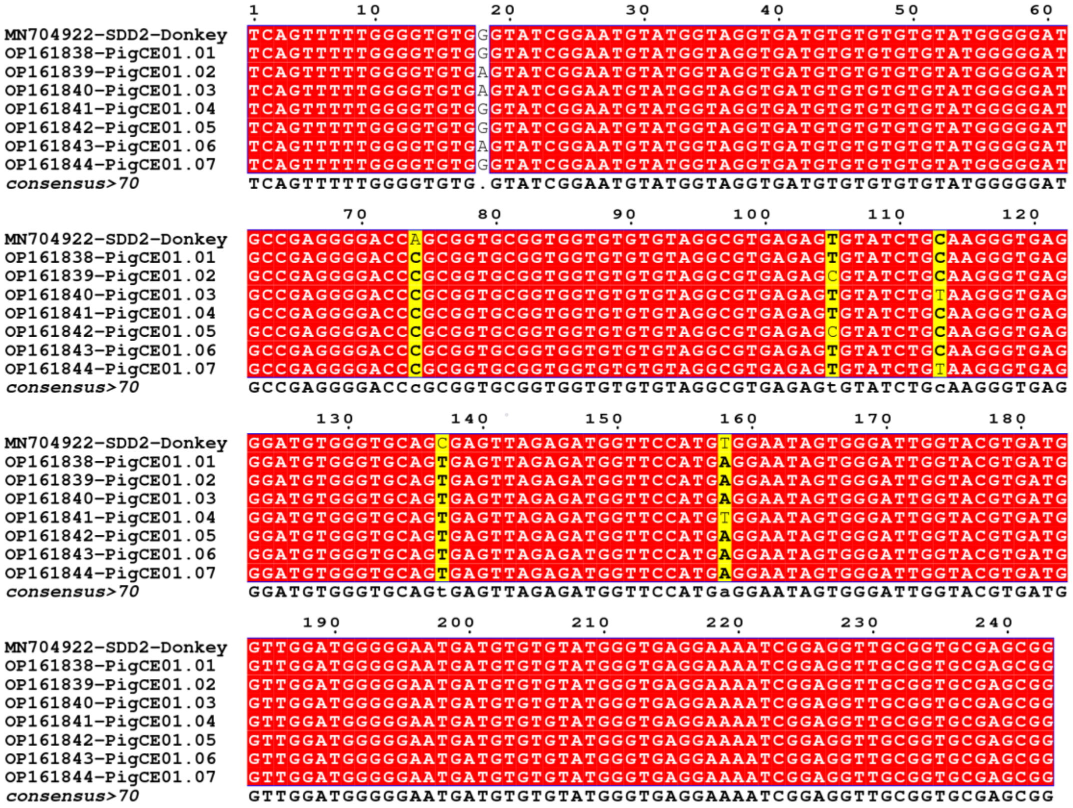

Supplementary Figure 1 (A). Multiple Alignment of PigCE01to the identify genotype SDD2

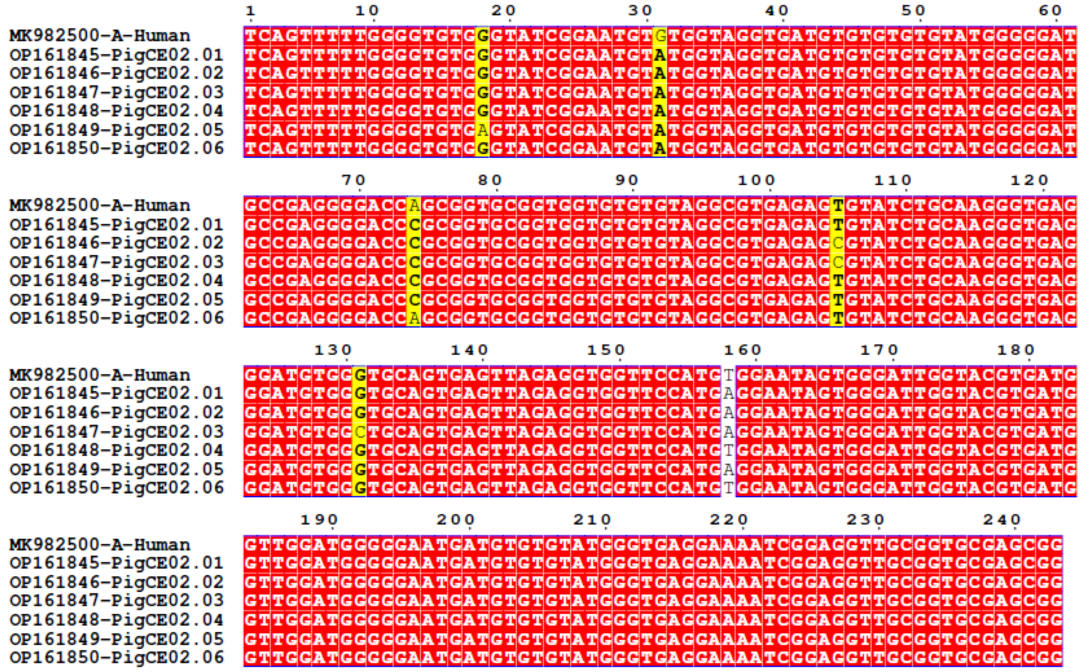

Supplementary Figure 1 (B). Multiple Alignment of PigCE02 to the identify genotype A

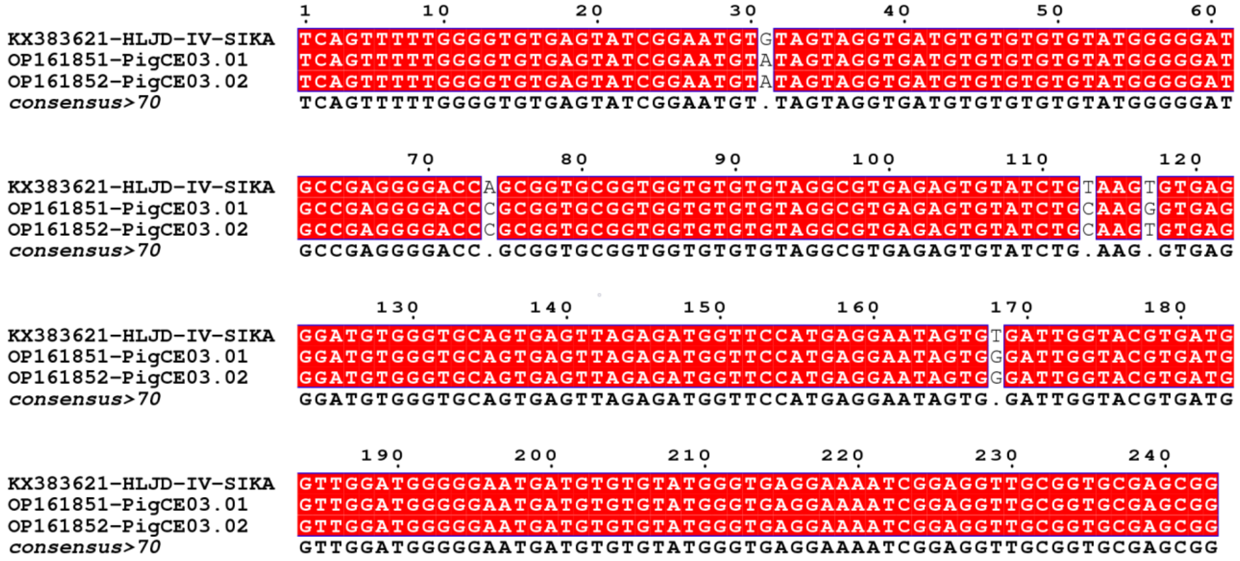

Supplementary Figure 1(**C**)**.** Multiple Alignment of PigCE03 to the identify genotype HLJD-IV

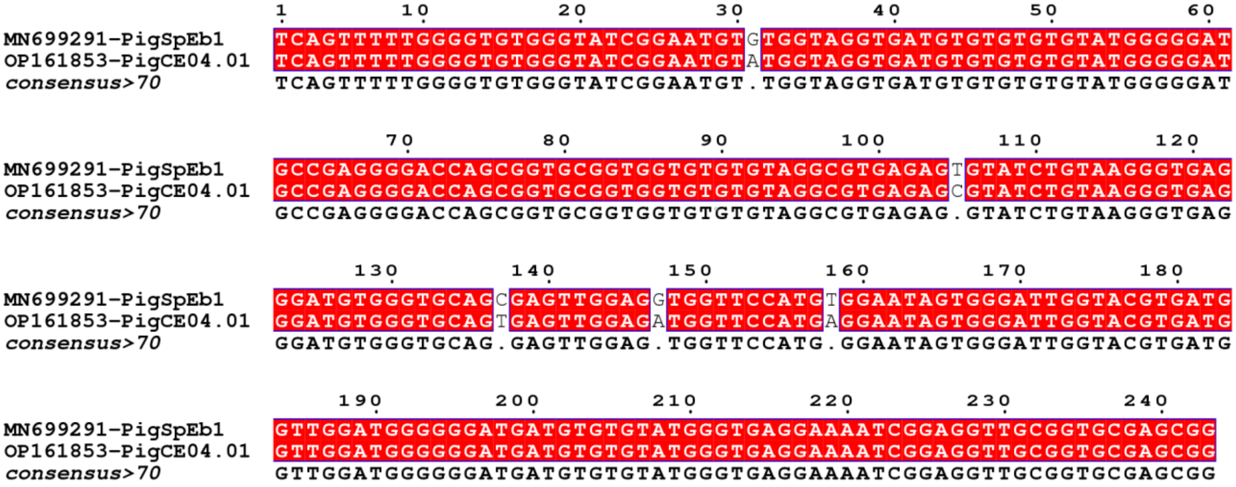

Supplementary Figure 1(D). Multiple Alignment of PigCE04 to the identify genotype PigSpEb1

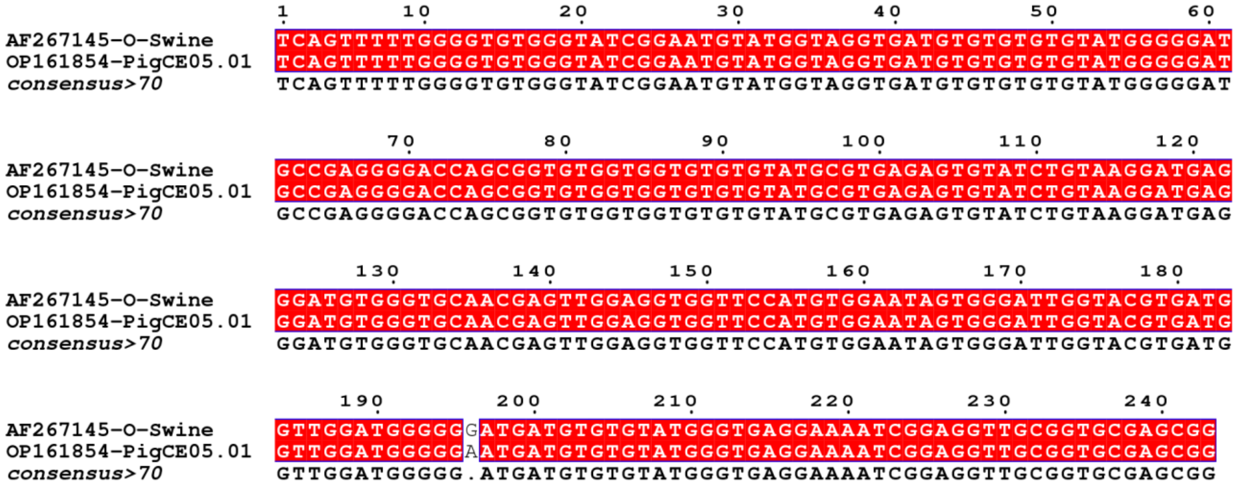

Supplementary Figure 1(E). Multiple Alignment of PigCE05 to the identify genotype O

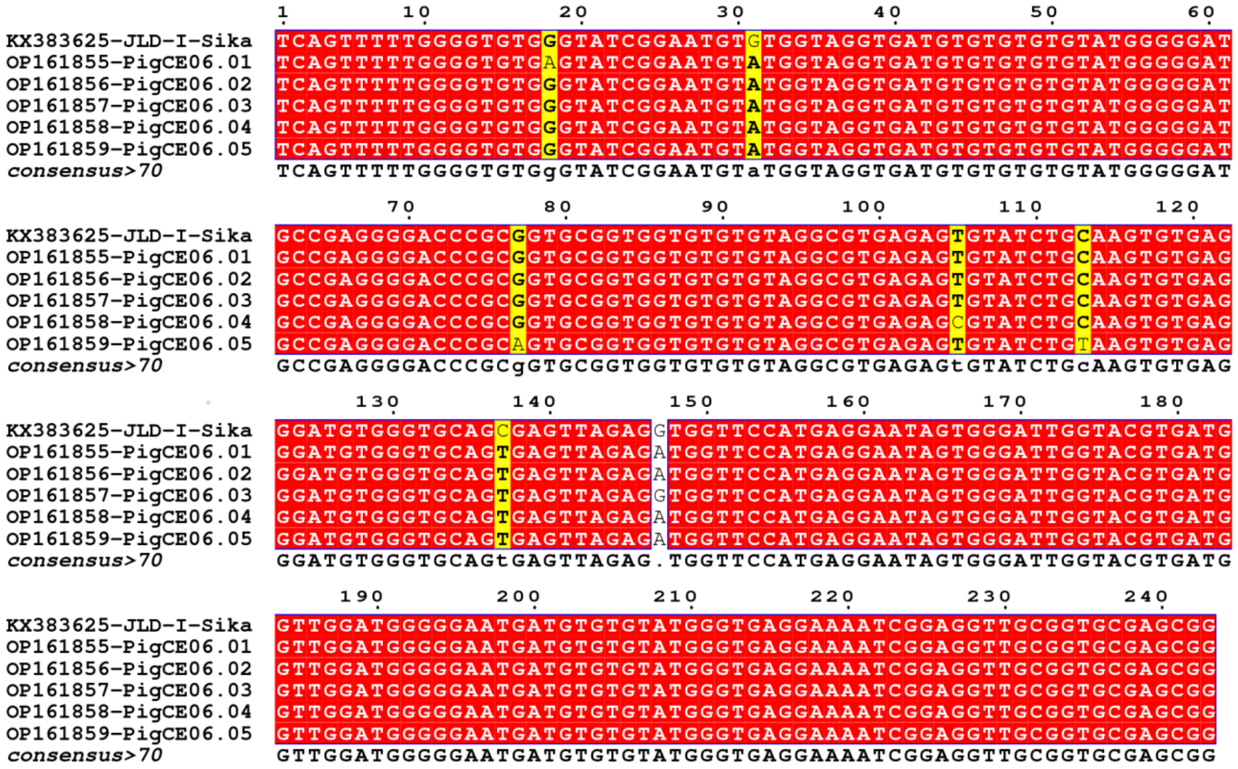

Supplementary Figure 1(F). Multiple Alignment of PigCE06 to the identify genotype JLD-I

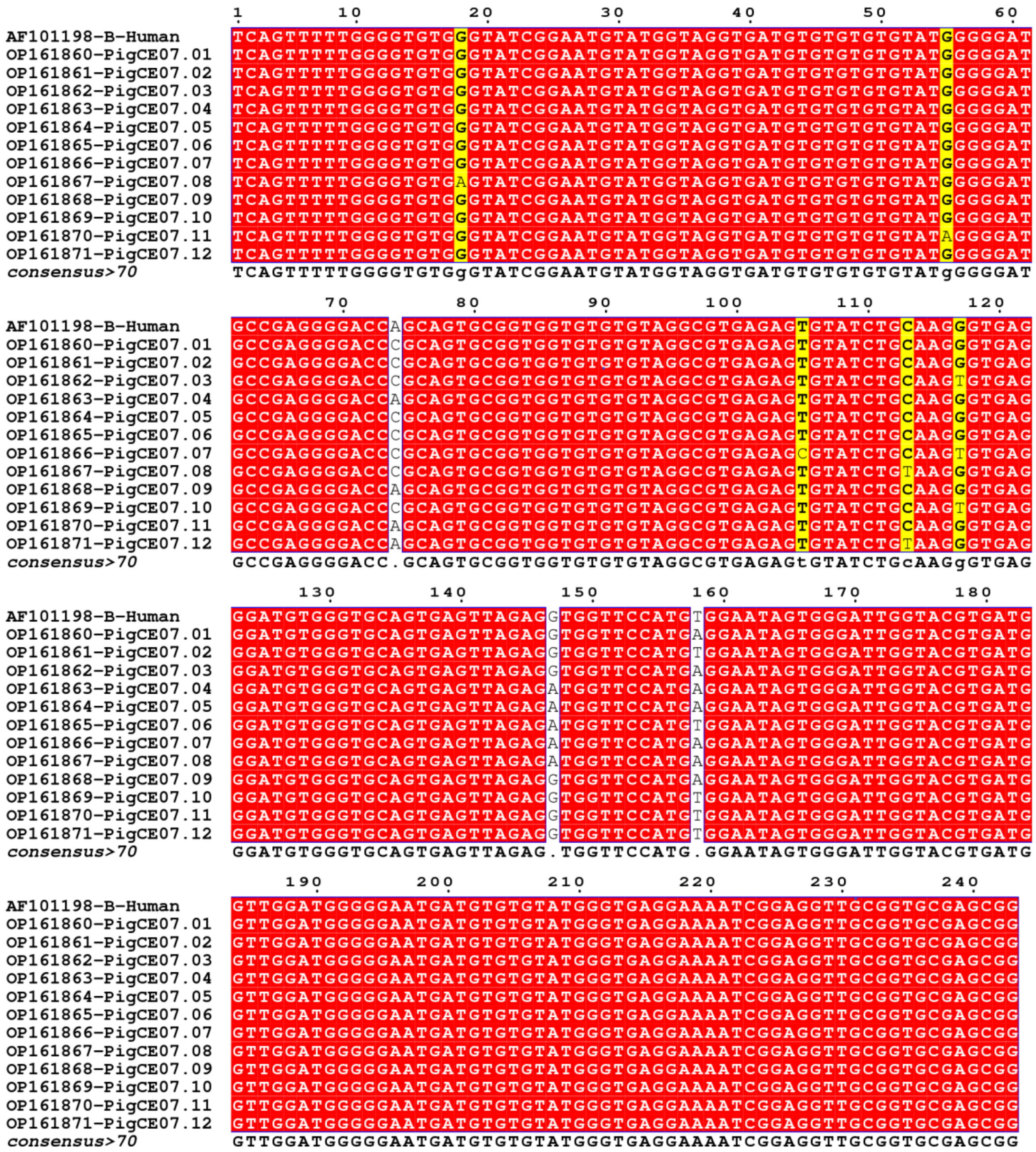

Supplementary Figure 1(G). Multiple Alignment of PigCE07 to the identify genotype B

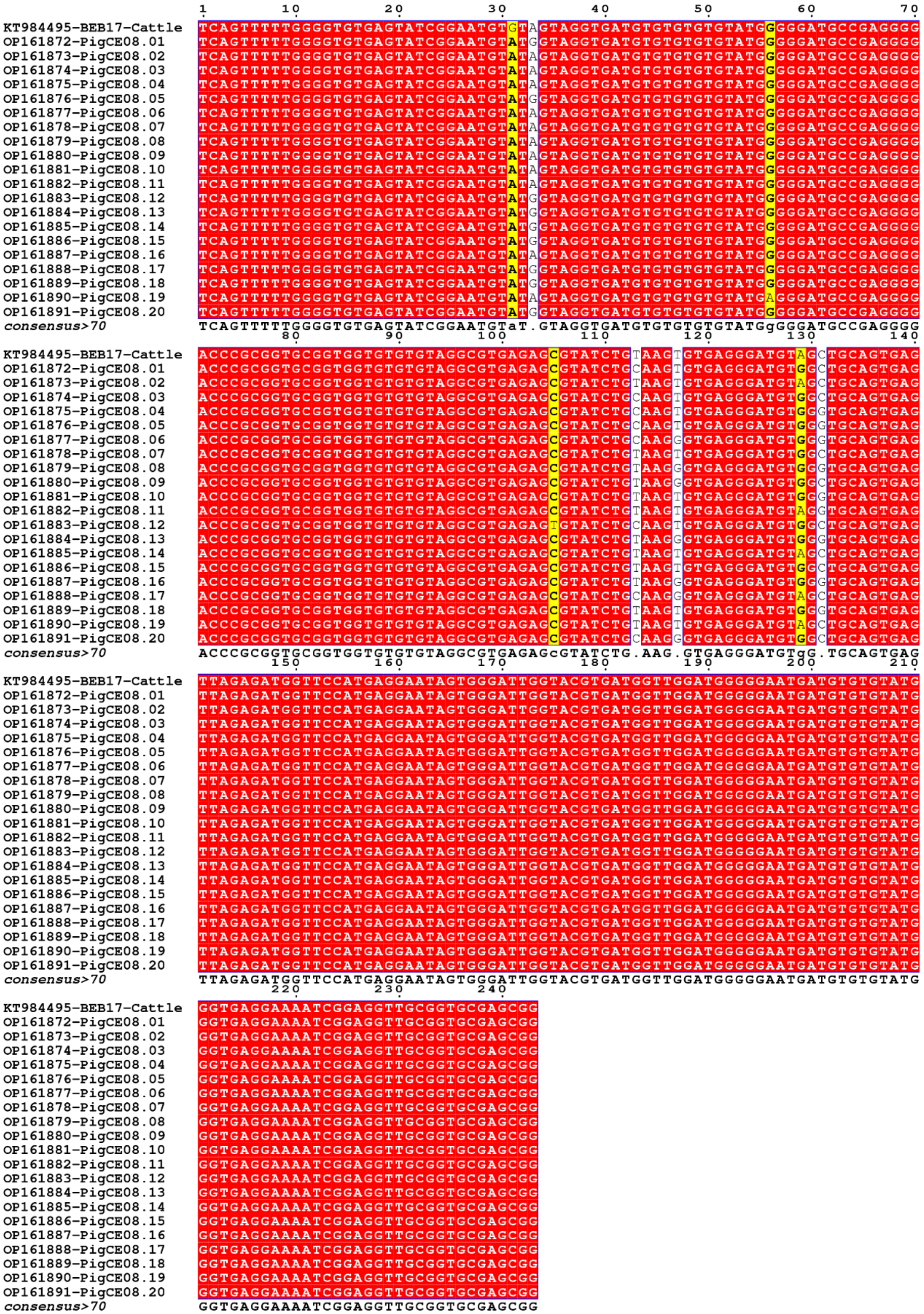

Supplementary Figure 1(H). Multiple Alignment of PigCE08 to the identify genotype BEB17

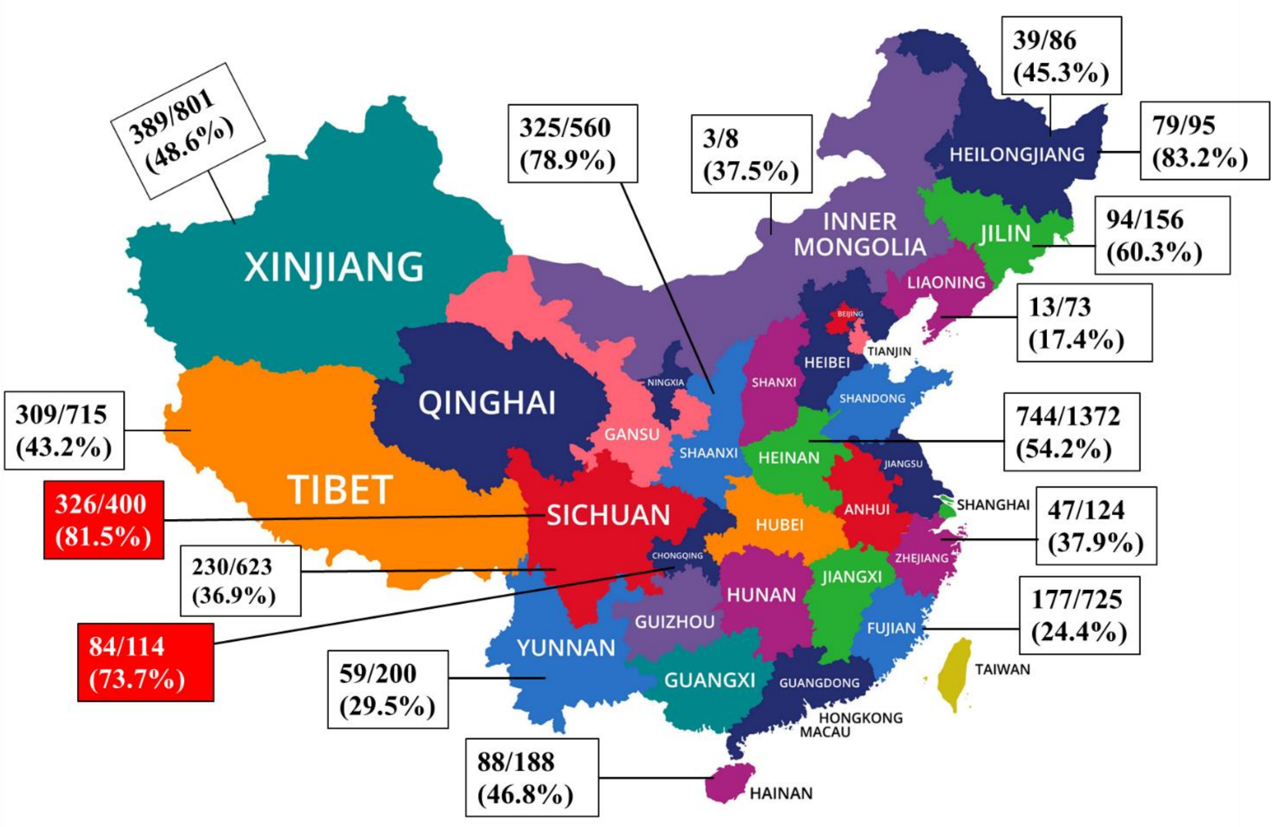

Supplementary Figure 2. Prevalence of *Enterocytozoon bieneusi* in China

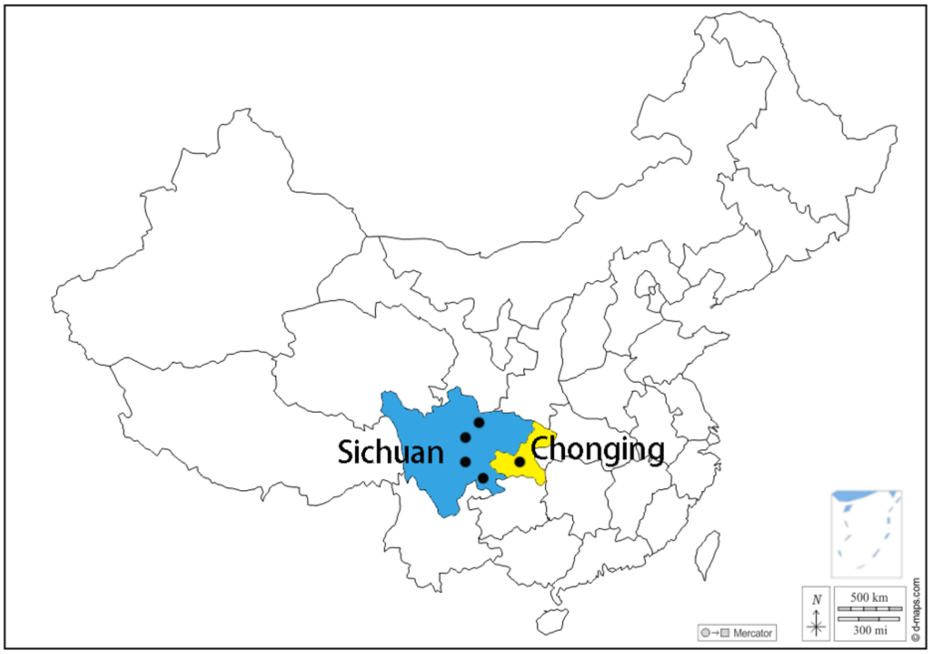

Supplementary Figure 3. Sampling sites of diarrheic pigs in Sichuan and Chongqing provinces.

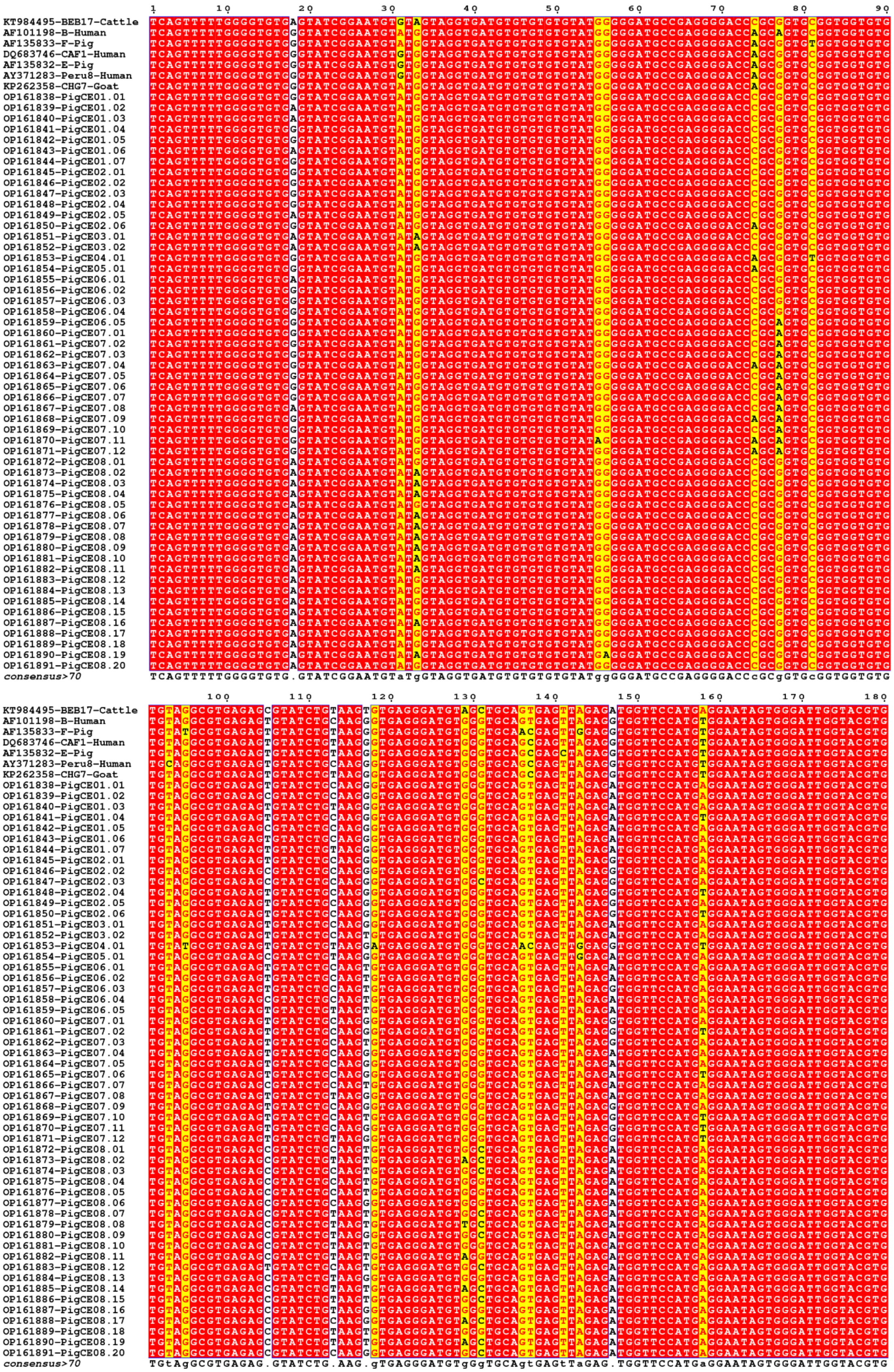
1 10 20 30 40 50 60 70 80 90
100 110 120 130 140 150 160 170 180
KT984495-BEB17-Cattle
AF101198-B-Human
AF135833-F-Pig
DQ683746-CAF1-Human
AF135832-E-Pig
AY371283-Peru8-Human
KP262358-CHG7-Goat
OP161838-PigCE01.01
OP161839-PigCE01.02
OP161840-PigCE01.03
OP161841-PigCE01.04
OP161842-PigCE01.05
OP161843-PigCE01.06
OP161844-PigCE01.07
OP161845-PigCE02.01
OP161846-PigCE02.02
OP161847-PigCE02.03
OP161848-PigCE02.04
OP161849-PigCE02.05
OP161850-PigCE02.06
OP161851-PigCE03.01
OP161852-PigCE03.02
OP161853-PigCE04.01
OP161854-PigCE05.01
OP161855-PigCE06.01
OP161856-PigCE06.02
OP161857-PigCE06.03
OP161858-PigCE06.04
OP161859-PigCE06.05
OP161860-PigCE07.01
OP161861-PigCE07.02
OP161862-PigCE07.03
OP161863-PigCE07.04
OP161864-PigCE07.05
OP161865-PigCE07.06
OP161866-PigCE07.07
OP161867-PigCE07.08
OP161868-PigCE07.09
OP161869-PigCE07.10
OP161870-PigCE07.11
OP161871-PigCE07.12
OP161872-PigCE08.01
OP161873-PigCE08.02
OP161874-PigCE08.03
OP161875-PigCE08.04
OP161876-PigCE08.05
OP161877-PigCE08.06
OP161878-PigCE08.07
OP161879-PigCE08.08
OP161880-PigCE08.09
OP161881-PigCE08.10
OP161882-PigCE08.11
OP161883-PigCE08.12
OP161884-PigCE08.13
OP161885-PigCE08.14
OP161886-PigCE08.15
OP161887-PigCE08.16
OP161888-PigCE08.17
OP161889-PigCE08.18
OP161890-PigCE08.19
OP161891-PigCE08.20
consensus>70 TCAGTTTTTGGGGTGTG.GTATCGGAATGTaTgGTAGGTGATGTGTGTGTGTATggGGGATGCCGAGGGGACCcGCgGTGcGGTGGTGTG
consensus>70 TGtAgGCGTGAGAG.GTATCTG.AAG.gTGAGGGATGTgGgTGCAgtGAGtTaGAG.TGGTTCCATGaGGAATAGTGGGATTGGTACGTG

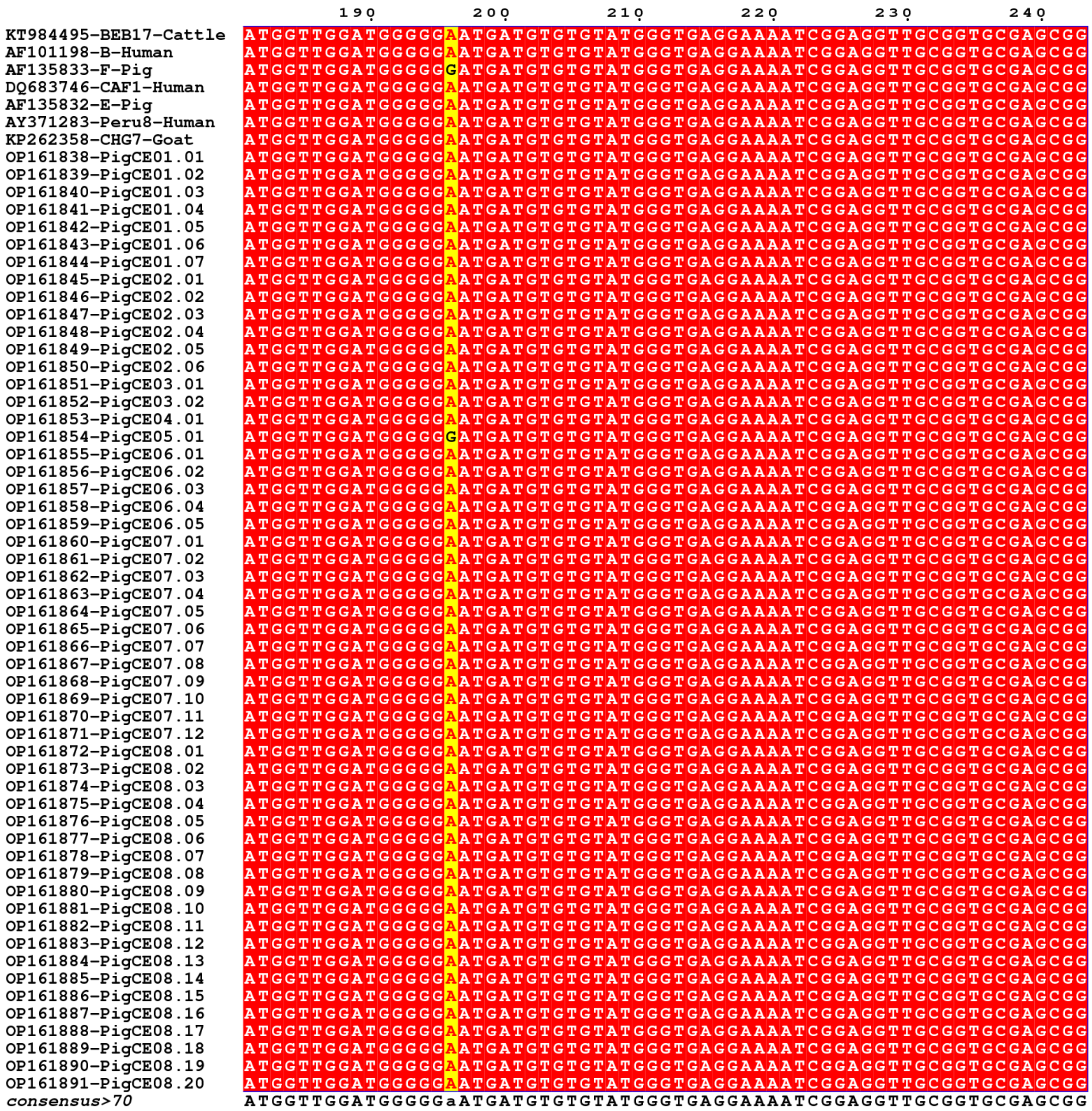

Supplementary Figure 4. Variation in the sequence of the ITS region of the rRNA gene in *Enterocytozoon bieneusi* isolates from diarrheic pigs. In this investigation, the ITS sequences of seven known genotypes (E, F, CHG1, Peru8, CAF1, B, and BEB17) and the 54 novel genotypes (PigCE01(7), PigCE02(6), PigCE03(2), PigCE04(01), PigCE05(01), PigCE06(5), PigCE07(12), and PigCE08(20) were aligned relative to the ITS sequence of genotype BEB17.
